# Comprehensive interdisciplinary treatment of an orbital lymphatic-venous malformation: a case report

**DOI:** 10.1515/iss-2025-0010

**Published:** 2025-06-30

**Authors:** Céline Tourbier, Bilal Msallem, Martin Thanh-Long Takes, Florian M. Thieringer, Lukas B. Seifert

**Affiliations:** 30262 Clinic of Oral and Cranio-Maxillofacial Surgery, University Hospital Basel, Basel, Switzerland; Department of Biomedical Engineering, Medical Additive Manufacturing Research Group (Swiss MAM), University of Basel, Allschwil, Switzerland; University Center for Orthopedics, Trauma and Plastic Surgery, Faculty of Medicine and University Hospital Carl Gustav Carus, TU Dresden, Dresden, Germany; Medizinisch Radiologisches Institut Zürich, Zurich, Switzerland

**Keywords:** lymphatic abnormalities, orbital diseases, eyelid diseases, plastic surgery procedures, sclerotherapy, case reports

## Abstract

**Objectives:**

Orbital lymphatic-venous malformations (LVMs) are rare congenital anomalies that can cause significant functional and aesthetic impairments.

**Case presentation:**

This case report details the comprehensive treatment of a 47-year-old male with a longstanding orbital combined slow-flow vascular malformation. Diagnostic evaluation included magnetic resonance imaging and a sonographically guided core needle biopsy, which confirmed a benign lymphatic-venous malformation. The treatment started with minimally invasive sclerotherapy to reduce lesion size, followed by surgical debulking to alleviate proptosis and mechanical restrictions. Finally, reconstructive surgery addressed residual eyelid deformities, including ectropion and ptosis. Post-treatment outcomes demonstrated stable visual function, improved eyelid position, and a significant enhancement in the patient’s aesthetic appearance and quality of life.

**Conclusions:**

The staged, multimodal approach underscores the importance of individualized treatment planning and interdisciplinary collaboration. This case highlights the potential for excellent functional and aesthetic outcomes in the management of complex orbital LVMs when nonsurgical and surgical techniques are strategically combined.

## Objectives

Orbital lymphatic-venous malformations (LVMs) are rare congenital or developmental anomalies involving both blood and lymphatic vessels within the orbit. According to the updated ISSVA classification (2025), vascular malformations are first divided based on flow characteristics into fast-flow and slow-flow lesions, with LVMs categorized as slow-flow combined vascular malformations​. Within the slow-flow group, malformations are further classified by vessel type into capillary, lymphatic, venous, or combined forms. LVMs are distinguished by the coexistence of lymphatic and venous components without one vessel type predominating [1]. These malformations are typically diagnosed in childhood or early adulthood and can be differentiated from other vascular anomalies through radiologic and histopathologic evaluation based on flow characteristics and vessel type ​[2], 3]. LVMs are challenging due to their mixed structure and potential for compressive symptoms within the confined orbital space. These can lead to complications such as proptosis, ptosis, and, in severe cases, compressive optic neuropathy [3], 4].

These malformations are usually low-flow, presenting as nonpulsatile, compressible masses that impact surrounding tissues through mass effect rather than active blood flow [4]. The functional impairment and aesthetic deformity caused by these lesions significantly affect a patient’s quality of life, underscoring the need for an interdisciplinary approach to management [4]. Treatment varies by malformation size, location, and symptom severity, with minimally invasive options like sclerotherapy or embolization often considered first-line treatments. Surgical intervention may sometimes be necessary for persistent or recurrent lesions that compromise visual function or cause aesthetic concerns [4], 5].

## Case presentation

A 47-year-old male presented with a 30-year history of a vascular malformation in the left orbit, initially characterized by intermittent swelling that spontaneously regressed in earlier years. In recent years, however, the malformation exhibited persistent growth, leading to proptosis, ectropion, and ocular dryness. A sonographically guided core needle biopsy confirmed the diagnosis of an LVM with no evidence of malignancy. Following the confirmation of the lesion’s benign nature, a series of sonographically guided sclerotherapy sessions were initiated to reduce lesion size and alleviate symptoms. The first session, conducted in September 2020, utilized 3 % polidocanol (Aethoxysklerol®, Kreussler & Co. GmbH, Wiesbaden, Germany) under sonographic guidance and was well tolerated. Subsequent sessions followed in January 2021 with 1 % polidocanol and in April 2021 with 3 % polidocanol, partially reducing the lesion size. Despite these interventions, symptoms persisted. In June and July 2021, more profound orbital sclerotherapy sessions were performed, offering transient symptom improvement. The treatment series concluded in September 2021 with a session using alcohol gel (DiscoGel®, Gelscom, Hérouville-Saint-Clair, France), targeting specific lesion areas. Post-treatment evaluation with magnetic resonance imaging (MRI) revealed a well-defined vascular lesion within the orbital space, displacing the globe superiorly without involvement of the optic nerve.

Due to the limited response to sclerotherapy, additional biopsies were performed, confirming the diagnosis of an LVM. Histopathology revealed a CD31+, D2–40+, and WT1+ immunophenotype, an irregular tunica media, and a reactive proliferation, thereby confirming the diagnosis. While sclerotherapy reduced lesion size and provided partial symptom relief, mechanical restrictions and discomfort persisted, necessitating consideration of alternative or supplementary treatments.

By March 2022, an ophthalmologic assessment revealed clear corneas, preserved visual acuity, and no double vision. However, a restricted downward gaze was observed, attributed to mechanical obstruction caused by the malformation.

## Surgical intervention

Given the limited success of nonsurgical therapies, the interdisciplinary team recommended surgical resection to achieve more effective symptom relief and improve aesthetic outcomes.

**Figure 1:**
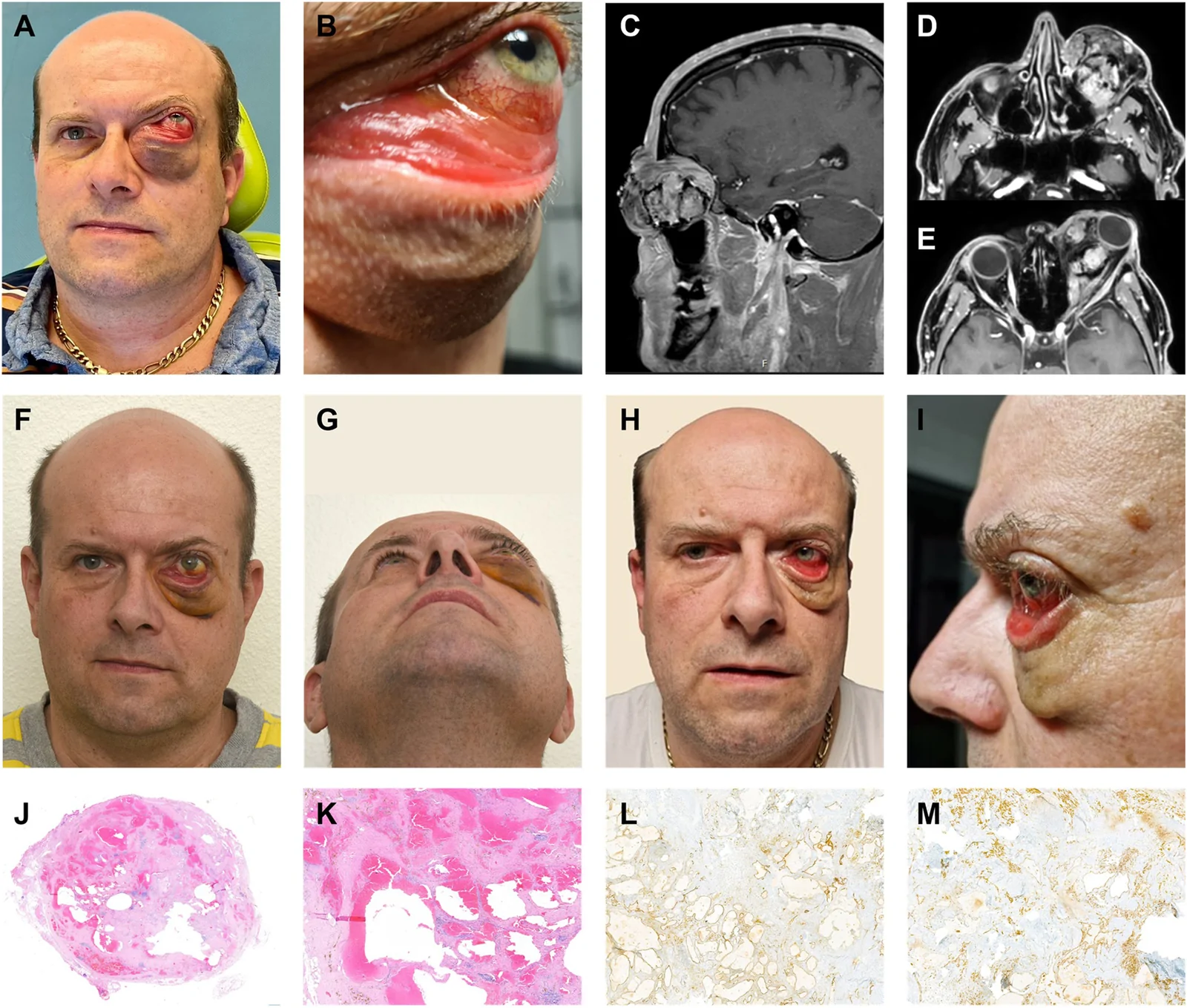
Clinical presentation, imaging, and histopathological analysis of a patient with a lymphatic-venous malformation (LVM) in the left orbital region: (A) frontal view and (B) close-up view of the left lower eyelid showing marked swelling due to the malformation after sclerotherapy. (C) Sagittal MRI reveals the malformation’s position in the left orbit, displacing the eye prior to surgical treatment. (D, E) Axial MRI scans provide further detail of the malformation’s extent within the left orbit. (F) Frontal view and (G) submental view of the face 1 week after tumor debulking. (H) Frontal view and (I) lateral view of the face 1 month postoperatively. (J) Overview of the excised tissue with hematoxylin and eosin staining. (K) High-magnification hematoxylin and eosin staining showing detailed tissue architecture. (L) Immunohistochemical staining for D2–40 positivity, a lymphoendothelial marker. (M) Immunohistochemical staining for CD31 positivity, an endothelial marker.

In November 2022, the patient underwent orbital tumor debulking under general anesthesia, with key intraoperative steps as follows: a subciliary incision was made, followed by careful dissection to access the orbital lesion (Figure 1). Vascular components near the optic nerve were removed meticulously to avoid critical structures. A Penrose drain was placed to minimize the risk of postoperative hematoma. Closure was performed with careful alignment of the eyelid to prevent postoperative deformities. The patient was treated with antibiotics, analgesia, antibiotic eye drops, and regular ophthalmologic monitoring. Histopathological analysis once again confirmed the benign lymphatic-venous nature of the lesion, with no evidence of malignancy.

## Reconstructive surgery

**Figure 2:**
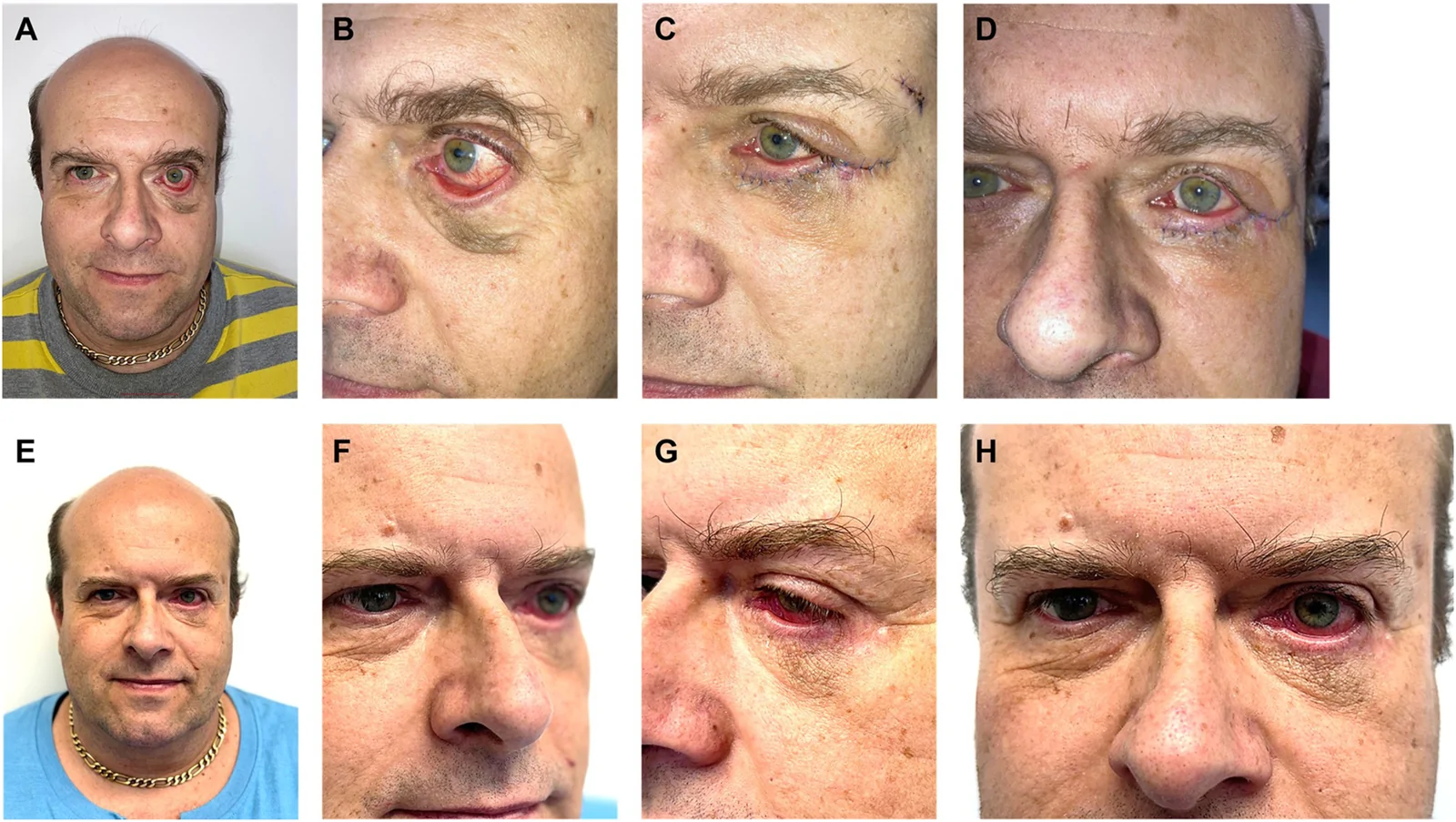
Blepharoplasty and lateral canthopexy: (A) frontal view and (B) oblique view of the patient 1 year after tumor debulking, demonstrating significant ectropion. (C) Oblique view and (D) close-up view 1 day after blepharoplasty and lateral canthopexy. (E) Frontal view, (F, G) oblique views, and (H) close-up view 6 weeks postoperatively, demonstrating improved facial symmetry and minimal scarring.

Following tumor excision, the patient experienced residual ectropion and lower eyelid ptosis, prompting reconstructive surgery to address both functional and aesthetic concerns. In February 2024, the patient underwent a lateral canthopexy to tighten the lower eyelid and a lower lid blepharoplasty to remove excess skin (Figure 2). Postoperative care, including cold compresses and antibiotic eye drops, facilitated recovery and led to a marked improvement in eyelid function and appearance.

Follow-up assessments from February to August 2024 demonstrated a sustained recovery. Visual function remained intact with no new deficits, and ocular motility improved with minimal restriction. The eyelid position showed full resolution of ectropion and ptosis, achieving satisfactory aesthetic results. The patient expressed satisfaction with functional and aesthetic outcomes and continued with annual follow-ups to monitor potential recurrence.

## Conclusions

As illustrated in this case, surgical intervention and subsequent reconstruction for complex orbital LVMs underscore the intricate nature of treating orbital malformations. The management approach, involving a combination of meticulous debulking surgery and reconstructive procedures, aligns with recommended practices in the literature for achieving functional relief and aesthetic satisfaction.

## Diagnostic and treatment considerations

Initial diagnostic imaging via MRI and sonographically guided core needle biopsy confirmed the benign nature of the malformation, aligning with similar cases in the literature where LVMs have been identified as benign but symptomatic entities [6].

Sclerotherapy, a minimally invasive technique, was selected as the first-line intervention for targeting the low-flow malformation in the periorbital region.

Commonly used agents include doxycycline, bleomycin, OK-432 (Picibanil), pingyangmycin, and sodium tetradecyl sulfate, all of which have demonstrated good overall response rates. Doxycycline is effective but can cause systemic side effects, while bleomycin shows good results with mostly minimal and transient complications [7], 8]. OK-432 is effective particularly for macrocystic lymphatic lesions but can occasionally cause facial nerve palsy probably due to the swelling of the lesion after treatment and nerval compression [8]. In the treatment strategy discussed, polidocanol was used initially due to its excellent safety profile; as a nonionic detergent, it induces endothelial damage and lesion regression with minimal inflammatory response​. Complication rates with polidocanol are low, making it highly appropriate for sensitive areas such as the periorbital region [8]. In contrast, ethanol is a potent sclerosant that promotes complete vascular obliteration; however, it is associated with higher rates of severe complications, including skin necrosis, ulceration, and nerve injury, and is generally reserved for refractory cases [8].

The outcomes of the sclerotherapy sessions suggest that sonographically guided sclerotherapy can achieve partial lesion size reduction and provide symptomatic relief, although it could not fully address long-term symptom control in this case. The 1 % polidocanol session was well-tolerated, consistent with findings regarding patient acceptance [9]. In the follow-up session, 3 % polidocanol achieved partial lesion reduction, consistent with reports that higher polidocanol concentrations may enhance lesion reduction but might not completely resolve symptoms [10]. Subsequent sessions in June and July 2021 targeted more profound orbital components of the lesion, resulting in temporary size reduction, an approach noted in the literature as beneficial for addressing complex lesions within deeper structures [11]. However, the continued presence of symptoms, mainly mechanical restrictions, suggests that sclerotherapy alone may be insufficient for comprehensive symptom relief.

In the final sclerosis session, gelified ethanol (DiscoGel®) was applied. This sclerosant has a very high safety profile as it is very viscous after contact to ionic liquids such as blood and stays inside the vessels. An MRI follow-up was planned for long-term assessment. This reflects the attempt to improve the efficacy of sclerotherapy for sustained outcomes [12]. While this method holds potential for certain lesion types, as indicated in prior studies, the persistent symptoms highlight the need to explore alternative or adjunctive treatments to achieve more lasting control.

## Surgical debulking and technique considerations

Orbital LVMs are often challenging to treat surgically due to their diffuse and infiltrative nature, which poses risks to critical orbital structures. Studies emphasize that surgery is usually reserved for cases with significant symptoms that are unresponsive to less invasive therapies. In our case, a subciliary approach allowed access to the orbital lesion, enabling careful resection near the optic nerve while protecting critical anatomy. This technique aligns with findings by Colletti et al. (2019), who noted that surgical resection, often involving complex intraoperative decisions to protect vital structures, remains a preferred approach for symptomatic orbital malformations when sclerotherapy alone is insufficient [6].

The use of an aspiration drain postsurgery can help manage the risk of postoperative complications such as hematoma formation and cystic recurrence, which are concerns due to the lesion’s propensity for bleeding [13].

## Reconstruction and functional outcomes

Postoperative reconstruction, including lateral canthopexy and lower lid blepharoplasty, was necessary to correct residual ectropion and ptosis resulting from both the malformation and surgical manipulation. Such reconstructive procedures are critical for restoring eyelid function and aesthetic symmetry, as highlighted by Nassiri et al. (2015), who discuss the aesthetic and functional benefits of targeted reconstructive surgery following orbital tumor debulking [5].

Reconstructive efforts postresection can substantially improve quality of life and patient satisfaction by addressing the functional and aesthetic sequelae of orbital malformations [5]. These techniques ensure enhanced symmetry, minimized scarring, and improved eyelid position, all achieved in this case.

This case illustrates the necessity of a tailored, interdisciplinary approach to managing complex orbital LVMs. In the rapidly evolving field of medicine, interdisciplinary collaboration assumes paramount importance, fostering the integration of knowledge from diverse medical specialties, including radiology, pathology, ophthalmology, and oral and maxillofacial surgery. Combining surgical debulking with reconstructive procedures can achieve substantial functional and aesthetic outcomes, making it a viable strategy when nonsurgical techniques do not provide sufficient relief.

Overall, surgical intervention with careful planning and reconstructive support is essential for achieving long-term symptom control and improved patient satisfaction. In similar cases, early consideration of surgical intervention combined with sclerotherapy and interdisciplinary planning may enhance outcomes. Tailoring the treatment strategy to the specific lesion behavior and patient presentation could optimize results and guide future management of complex orbital LVMs.
